# Safeguarding in dance as a public health issue: a systems-level policy framework

**DOI:** 10.3389/fpubh.2026.1919552

**Published:** 2026-08-31

**Authors:** Jennifer Cumming, Mary L. Quinton

**Affiliations:** 1 School of Sport, Exercise and Rehabilitation Sciences, University of Birmingham, Edgbaston, United Kingdom; 2 Institute for Mental Health, University of Birmingham, Edgbaston, United Kingdom

**Keywords:** biopsychosocial model, dance, health equity, mental self-harm, public health policy, safeguarding in sport, systems thinking

## Abstract

Safeguarding in dance is an under-recognized public health policy issue, characterized by inconsistent implementation and limited regulation. Drawing on public health frameworks, we conceptualize safeguarding-related harms through a biopsychosocial lens, encompassing physical, psychological, and social impacts. These harms are shaped by interacting factors across individual, interpersonal, organizational, and structural levels. The absence of systemic surveillance and regulatory oversight limits prevention, early intervention, monitoring, and evaluation of safeguarding practices. We propose six complementary policy actions: national surveillance systems, safeguarding audits, a quality mark, clear reporting frameworks, funding-linked accountability, and trauma- and violence-informed care. Together, these actions provide a coordinated, systems-level response, improving accountability, and promoting safe, healthy, and more equitable dance participation.

## 1 Introduction

Safeguarding in sport and performance contexts is increasingly recognized as a public health issue, reflecting growing awareness that participation environments can expose individuals to significant harm (1, 2). However, safeguarding in dance remains comparatively under-recognized within public health policy, despite emerging evidence of its wide-ranging impacts (3, 4). The World Health Organization (WHO) defines violence and abuse as public health problems requiring population-level prevention strategies (5), a framing now advocated in sport governance (1, 6). Applying this lens to dance highlights safeguarding failures, including direct physical harm, sexual coercion, and inappropriate touch disguised as instruction, as systemic risks to population health (7–9). These risks extend beyond isolated incidents, reflecting broader cultural and structural conditions within dance (3).

A public health approach requires understanding of safeguarding-related harms through a biopsychosocial framework, recognizing that maltreatment can affect physical, psychological, and social wellbeing (2, 10). In dance, aesthetic demands, early specialization, and the normalization of pain and obedience act as chronic stressors, contributing to physical (e.g., injuries, eating disorders), psychological (e.g., anxiety, trauma), and social wellbeing (e.g., identity loss, social exclusion) (9, 11–15). Despite increasing policy attention, safeguarding in dance remains inconsistently implemented. While many organizations report having formal policies, monitoring is limited and gaps in practice are evident, with nearly half of UK dance organizations reporting that they do not address all relevant safeguarding concerns (16, 17). This disconnect between policy and practice constrains prevention, limits early intervention, and undermines trust in safeguarding systems (18).

**Figure 1:**
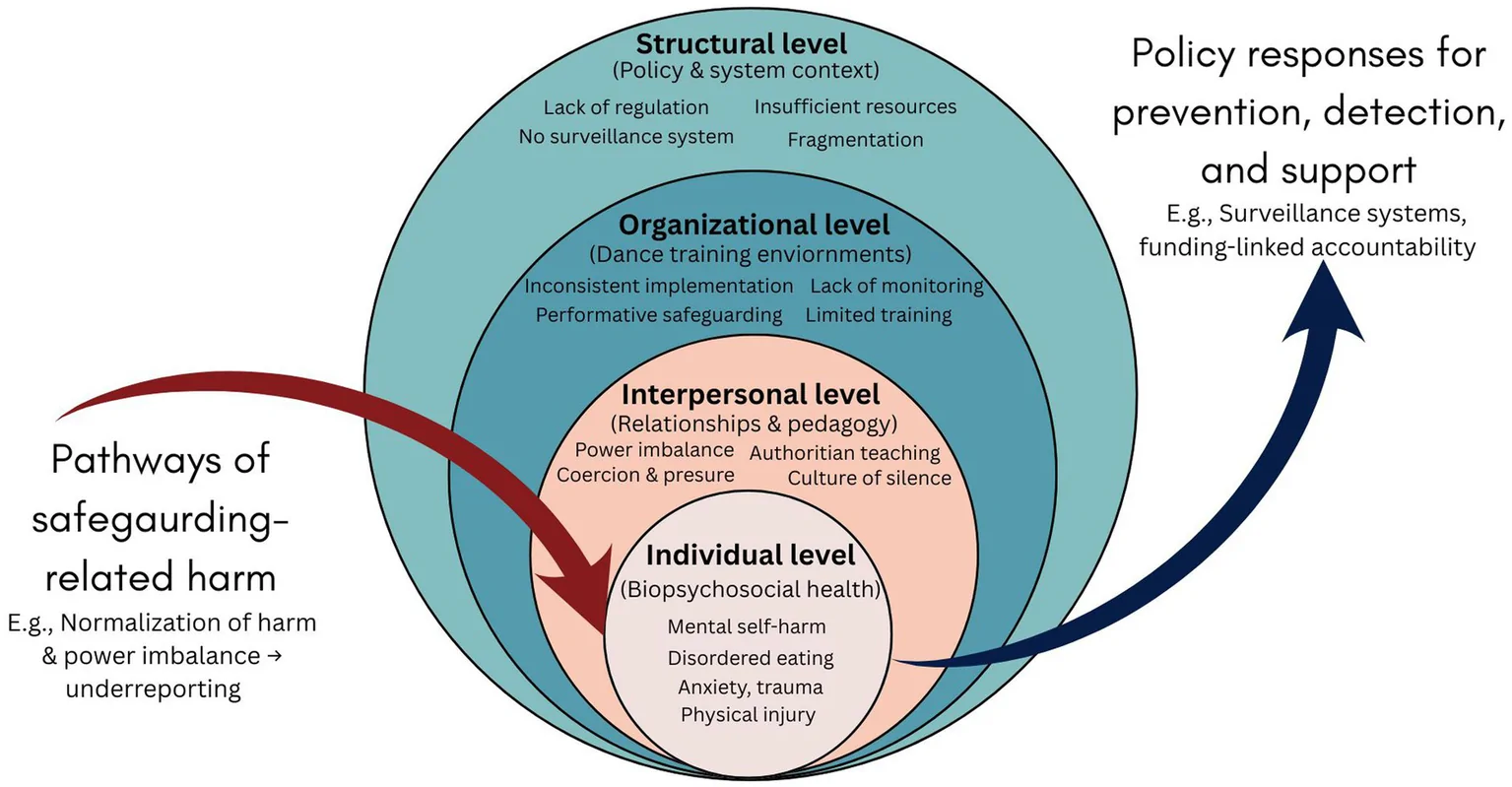
A systems-based public health model of safeguarding-related harm and policy response in dance. This figure illustrates how safeguarding in dance operates as a multi-level public health system, encompassing interacting determinants across individual, interpersonal, organizational, and structural levels. These interacting factors shape health outcomes and contribute to safeguarding-related harms at the individual level, including physical, psychological, and social impacts. The model also highlights how coordinated policy responses, such as surveillance and funding-linked accountability, can be applied across these levels to strengthen prevention, early detection, and response.

A systems thinking approach has been increasingly applied to safeguarding in sport and performance contexts, drawing on socioecological and public health frameworks (3, 19, 20). As outlined in Table 1 and illustrated in Figure 1, safeguarding-related harms and health outcomes in dance are shaped by interacting factors across individual, interpersonal, organizational, and structural levels. Interventions focused solely on individual resilience or injury rehabilitation are therefore insufficient. A systems perspective highlights how institutional policies, power dynamics, and cultural norms collectively shape dancer wellbeing (1, 3, 4, 16, 17, 20). This policy brief therefore applies a systems-based public health framework to safeguarding in dance and proposes coordinated policy actions to improve prevention, accountability, and equity.

**Table 1:** Multi-level determinants of safeguarding-related harm in dance.

Level	Key risk factors	Health implications	Policy leverage
Individual	Early specialization (43) Maladaptive perfectionism, low self-esteem, docility, and obedience (43) Lack of awareness of what constitutes reportable harm (28, 41, 44) Vulnerabilities tied to social identities (e.g., gender, race, and socioeconomic status) (12)	“Mental self-harm” (e.g., obsessive self-deprecating thoughts), eating disorders, and body dysmorphia (9, 24) Physical injuries from ignoring pain limits (13, 14) Anxiety, depression, PTSD, and loss of identity (9, 15, 43, 45) Long-term physical and mental health risks (7, 46)	Safeguarding literacy and education for dancers (3, 43) Provision of mental skills programs and TVIC support to empower dancers and build resilience (3, 43)
Interpersonal	Power imbalance and authoritarian pedagogy (11, 21) Use of hurtful comments, body shaming, and physical expressions of frustration by teachers (22) Grooming, coercion, favoritism, and the silent complicity of bystanders (peers and parents) (3, 8) Fear of reprisal or damaging career prospects (7)	Relational trauma, social exclusion, and marginality (7, 43, 46) Physical injuries resulting from unsafe pedagogical practices or forced manipulations (8, 15, 22) Reduced help-seeking (8, 13, 24)	Codes of conduct that define boundaries, such as safe touch policies and limits on mentor/apprentice relationships (3) Training instructors in TVIC and student-centered, “corrective” pedagogies to prevent re-traumatization (3, 10) Tracking “low-level concerns” to intercept harmful behaviors (22)
Organizational	Lack of independent monitoring and reporting mechanisms (33, 36) Performative safeguarding (33) Unregulated recruitment of teachers without pedagogical or safeguarding certification (3, 33)	Hidden burden of harm where maltreatment escalates unnoticed (4, 15) Worsening of physical injuries and mental health crises due to medical mismanagement or lack of support (4, 7, 15, 45) Inconsistent protection (3)	Safeguarding audits to ensure accountability (41) Safe, anonymous, and independent reporting pathways free from conflicts of interest (43) Establishing multidisciplinary referral pathways so staff refer dancers to qualified medical/psychological professionals (43)
Structural	Normalization of “suffering for the art” to achieve success (7, 11, 13, 21, 23) Extreme aesthetic demands rooted in Eurocentric ideals (12) Sector-wide safeguarding lacking coordination and regulatory oversight (3)	Systemic and cyclic transmission of trauma across generations of dancers and teachers (9) Widespread public health failures that systematically compromise the biopsychosocial well-being of the dance population (2)	Establishing a national safeguarding quality mark or tiered accreditation system (47) Linking public funding directly to demonstrated safeguarding compliance and implementation (32) Implementing national surveillance systems to gather baseline epidemiological data to proactively guide public health interventions (36, 41)

TVIC, trauma- and violence-informed care.

## 2 Policy problem

### 2.1 Cultural normalization and health risk

A central challenge in dance safeguarding is the normalization of harmful practices. Traditional pedagogical models emphasize discipline, hierarchy, and obedience, often blurring the boundary between rigorous teaching methods and harmful behavior (11, 21). Practices such as public humiliation, body shaming, and excessive physical demands may be embedded within training environments and rationalized as necessary for artistic excellence (7, 22). This cultural context reduces the likelihood that harm is recognized or challenged, creating a pervasive culture of silence and deference amongst dancers (15, 23).

### 2.2 Intrapersonal harm and hidden health burden

Safeguarding in dance must also account for intrapersonal forms of harm that are often under-recognized within policy frameworks (24). Dancers frequently experience disordered eating, over-exercising, negative self-evaluation, and other forms of “mental self-harm” shaped by extreme aesthetic and performance pressures (9, 25). These behaviors contribute to a hidden burden of harm, with severe implications for both mental and physical health. However, because these practices are often rewarded as signs of dedication, they are normalized within dance culture and therefore overlooked or minimized in safeguarding systems (15, 26).

### 2.3 Underreporting and barriers to help-seeking

The effectiveness of safeguarding is further limited by underreporting of concerns. The culture of silence is reinforced by power imbalances and discourages dancers from disclosing harm or seeking support (7). Reports suggest that dancers feel unable to raise concerns within their organizations due to fear of retaliation, such as losing roles or damaging future career prospects (4, 15). In other performance contexts, this fear of backlash leads to harms going unreported or unaddressed, thereby limiting opportunities for early intervention and allowing abusive practices to persist unchecked (15, 27, 28).

### 2.4 Organizational gaps in implementation and training

Even where safeguarding policies exist, implementation is inconsistent. Many organizations lack systematic monitoring and evaluation, and safeguarding training is limited in scope and duration (16). Teacher education in dance is typically unregulated, with many instructors entering teaching roles without formal training in pedagogy or safeguarding (3, 4). This contributes to the reproduction of harmful practices across generations, as new teachers may default to pedagogical approaches they experienced, including authoritarian teaching and normalized excessive physical training (11, 21, 23, 29).

### 2.5 Structural weaknesses and absence of surveillance

At a structural level, the dance sector does not have agreed standards or an infrastructure for safeguarding, and regulatory oversight is limited. Unlike other areas of sport and education, there is no consistent system of accountability or data collection related to safeguarding and organizations are rarely (if ever) monitored by neutral, third-party evaluators (30). Consequently, accountability is left to staff within the organization, creating significant conflicts of interest that may allow harmful cultures to operate undetected (3, 31). The absence of systematic surveillance means that the prevalence and determinants of safeguarding harms also remain poorly understood. Without reliable data, it is difficult to determine whether the scale of the problem is increasing or decreasing (32), reducing capacity to identify priorities, allocate resources, or evaluate policy impact. In turn, this limits the effectiveness of safeguarding efforts and risks performative approaches, whereby policies are adopted for visibility and credibility without meaningful implementation (33).

## 3 Policy options and implications

### 3.1 Establish national surveillance of safeguarding harms in dance

Establishing national surveillance systems would enable routine collection of data on safeguarding concerns, including psychological harm, disordered eating, and abuse. This would support the identification of high-risk environments and vulnerable groups, inform prevention strategies, and provide a basis for evaluating policy impact. In the absence of surveillance, safeguarding remains largely dependent on individual reporting, placing disproportionate responsibility on dancers (7, 34). Integrating surveillance into safeguarding systems is therefore essential to enable proactive, evidence-based approaches to prevention. To avoid connotations of excessive discipline or control associated with the term “surveillance”, Kerr and Kerr’s (35) recommendation of an “oligopticon” model (i.e., collecting narrow slices of information from diverse, dispersed sources) should also be considered in dance.

### 3.2 Mandate safeguarding audits

While surveillance provides population-level insight, safeguarding audits are necessary to improve accountability at the organizational level and address inconsistent implementation and evaluation of safeguarding policies and limited evaluation of their effectiveness. Mandating regular safeguarding audits would require dance organizations to systematically review their policies, practices, and outcomes. It would help to address performative safeguarding by conveying that the mere existence of safeguarding policy on paper is not sufficient (18, 33). Audits could incorporate a standardized set of indicators related to training, reporting, and organizational culture, providing a mechanism for identifying gaps and monitoring progress (36). By requiring organizations to report against standardized, evidence-based metrics, the dance sector can benchmark progress and clearly identify systemic weaknesses. Data from these evaluations can then be used to drive continuous, dynamic policy improvement, supporting continuous improvement and alignment with public health principles quality assurance and evaluation (36).

### 3.3 Establish a tiered safeguarding quality mark

The absence of consistent standards across the dance sector contributes to wide variation in safeguarding practice. Without clear benchmarks, it is difficult to distinguish between organizations that are effectively implementing safeguards and those that are not (3, 36). A safeguarding quality mark would provide a standardized framework for safeguarding, defining minimum expectations for policy, training, implementation, and review. A tiered approach (e.g., bronze, silver, and gold) goes a step further to acknowledge that organizations are at different stages in their safeguarding journey. It would define minimum standards whilst also incentivizing continuous improvement, enabling parents to check a dance organization’s safeguarding status before enrolling their child in dance training (37). Accreditation would also promote greater transparency and support the dissemination of best practice. In public health terms, this represents a move toward standardization of prevention environments, reducing variability in exposure to risk.

### 3.4 Clarify reporting thresholds and procedures

Underreporting remains one of the most significant barriers to effective safeguarding (28, 38). Developing clear, sector-specific guidance on reporting thresholds and procedures co-produced with safeguarding experts and individuals with lived experience would improve consistency and accessibility. This includes defining what constitutes a safeguarding concern, outlining when escalation is required, and establishing safe, independent reporting pathways. Dance organization should be mandated to track low-level concerns to better detect patterns, recognize maltreatment behaviors, and proactively implement early-warning interventions before serious harm occurs (39). Strengthening reporting systems to support early detection and intervention is central to effective public health prevention.

### 3.5 Link funding to safeguarding implementation

With policies alone not being sufficient to drive change, linking public funding to demonstrated safeguarding implementation would provide a further structural lever to incentivize compliance (40). For those organizations who receive public funding, requiring evidence of safeguarding training, monitoring, and reporting systems as a condition of this funding would align institutional priorities with health and wellbeing outcomes. A caveat would be that compliance could not be self-reported, as this might lead to organizations being highly motivated to exaggerate their progress or simply “tick a box” (40). Ideally, compliance would be measured through mandatory, standardized audits conducted by a neutral, independent oversight body (41). This approach would address safeguarding as a structural determinant of health, reinforcing its importance at the system level (2).

### 3.6 Promote trauma- and violence-informed care and practice

Embedding trauma- and violence-informed care (TVIC) with dance organizations would improve recognition of harm, reduce re-traumatization, and support recovery (42). This includes training staff and ensuring access to multidisciplinary support, including psychological care. Such approaches are particularly important in addressing hidden harms, including mental self-harm and disordered eating (24). By prioritizing safety, trust, and agency, TVIC can help restore autonomy and support meaningful engagement in dance (36).

## 4 Actionable recommendations

To address safeguarding in dance as a public health issue, coordinated action is required across multiple levels of the system. Table 2 summarises the recommended policy actions, alongside their underlying mechanisms and expected outcomes, illustrating how each intervention contributes to prevention, detection, and response.

**Table 2:** Actionable policy recommendations.

Policy	Actionable recommendations	Mechanisms	Outcomes
Develop national surveillance systems for safeguarding harms in dance	Establish systems for the routine collection and analysis of data on safeguarding concerns, including psychological harm, disordered eating, and abuse.	Enables identification of prevalence, trends, and at-risk groups.	Supports evidence-based policymaking, prioritization of interventions, and evaluation of impact.
Mandate regular safeguarding audits for dance organizations	Require organizations, particularly those receiving public funding, to undertake periodic audits of safeguarding policies, practices, and outcomes.	Provides structured monitoring of implementation and organizational culture.	Improves accountability, transparency, and continuous quality improvement.
Establish a national safeguarding quality mark	Develop an accreditation system defining minimum standards for safeguarding policy, training, implementation, and review.	Standardizes expectations and benchmarks performance across the sector.	Reduces variation in safeguarding practice and incentivizes improvement.
Standardize reporting frameworks and thresholds	Provide clear, sector-specific guidance on what constitutes a safeguarding concern and when reporting or escalation is required, supported by accessible and independent reporting pathways.	Reduces ambiguity and barriers to disclosure.	Improves early detection, intervention, and access to support.
Link public funding to safeguarding implementation	Require organizations to demonstrate evidence of safeguarding implementation, including training, monitoring, and reporting systems, as a condition of funding.	Aligns organizational incentives with health and safeguarding priorities.	Strengthens compliance and embed safeguarding as a core organizational responsibility.
Embed TVIC and practice	Mandate training and organizational approaches that recognize and respond to the impacts of harm, ensuring safe, supportive, and inclusive environments.	Improves recognition of harm, reduces re-traumatization, and supports engagement.	Enhances access to support, recovery, and equitable participation.

TVIC, trauma- and violence-informed care; mechanisms = how the policy is predicted to lead to changes in safeguarding; outcomes = what are the anticipated changes as a result of the policy.

## 5 Conclusion

This policy brief contributes to current debates by reframing safeguarding in dance as a public health issue and by identifying a set of complementary policy actions that collectively address prevention, detection, and response. Central to this approach is the need to strengthen surveillance, accountability, and trauma-informed practice, alongside efforts to standardize and embed safeguarding across the sector. Taken together, these recommendations offer a pathway towards more effective and equitable safeguarding systems. Without coordinated, system-level responses, safeguarding efforts are likely to remain poorly integrated and inconsistently implemented, limiting their capacity to prevent harm and protect dancer wellbeing.
